# Guanidine-Containing Antifungal Agents against Human-Relevant Fungal Pathogens (2004–2022)—A Review

**DOI:** 10.3390/jof8101085

**Published:** 2022-10-15

**Authors:** Simon D. P. Baugh

**Affiliations:** Fox Chase Chemical Diversity Center, Inc., 3805 Old Easton Rd., Doylestown, PA 18902, USA; sbaugh@fc-cdci.com

**Keywords:** antifungal agent, guanidine, small molecule, steroid, polymer, metal complex, sesquiterpene, natural product, polypeptide

## Abstract

The guanidine moiety is typically a highly basic group, and can be found in a wide variety of drugs, such as zanamivir (Relenza) and metformin (Fortamet), as well as in biologically active compounds for numerous disease areas, including central nervous system (CNS) diseases and chemotherapeutics. This review will focus on antifungal agents which contain at least one guanidine group, for the treatment of human-related fungal pathogens, described in the literature between 2004 and 2022. These compounds include small molecules, steroids, polymers, metal complexes, sesquiterpenes, natural products, and polypeptides. It shall be made clear that a diverse range of guanidine-containing derivatives have been published in the literature and have antifungal activity, including efficacy in *in vivo* experiments.

## 1. Introduction

Fungal infections are a growing problem in healthcare, and are associated with high mortality rates [1,2]. The available options for treating invasive fungal infections are severely limited, with there being only five classes of agents available for their treatment: polyenes (e.g., amphotericin B), echinocandins (e.g., micafungin), azoles (e.g., posaconazole), 5-flucytosine in combination with amphotericin B, and the most recent addition, ibrexafungerp. There are multiple issues with the available antifungal agents, including resistance to the drugs, drug–drug interactions, toxicity, and limited efficacy in the treatment of disseminated fungal infections. Thus, there is a substantial need for novel antifungal agents, preferably with different mechanisms of action compared to the current options.

**Figure 1:**
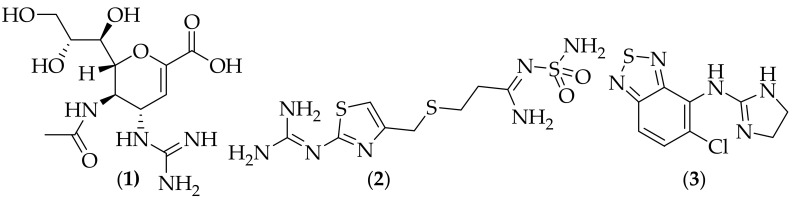
Structures of representative guanidine-containing approved drugs. zanamivir (Relenza) (**1**), famotidine (Pepcid), (**2**), and tizanidine (Zanaflex) (**3**).

The guanidine group is found in a broad range of drugs, including the anti-influenza drug zanamivir (Relenza) (**1**), the stomach acid reduction drug famotidine (Pepcid), (**2**) and the muscle relaxant tizanidine (Zanaflex) (**3**), Figure 1 [3]. The guanidine moiety has also been incorporated into a wide variety of biologically active compounds, including central nervous system (CNS) active analogs, antithrombotic agents, and chemotherapeutic agents [4,5]. As such, it is clear that it is quite reasonable to seek novel drug substances that incorporate a guanidine group.

Guanidine-containing compounds can also be isolated from natural sources [6,7], and natural products offer a variety of active antifungal agents, [8,9,10]. Among those natural products are a number that include a guanidine functionality-many of those were covered by an extensive review by Berlinck and Romminger, [7], and will not be included in the present review. This review will cover guanidine-containing small molecules, steroids, polymers, metal complexes, sesquiterpenes, natural products, and polypeptides, with antifungal activity against human-relevant fungal species, published between 2004 and 2022. The papers will predominantly be reviewed chronologically, but with related publications grouped together [11].

### 1.1. Guanidine-Containing Antifungal Agents 2004–2007

**Figure 2:**
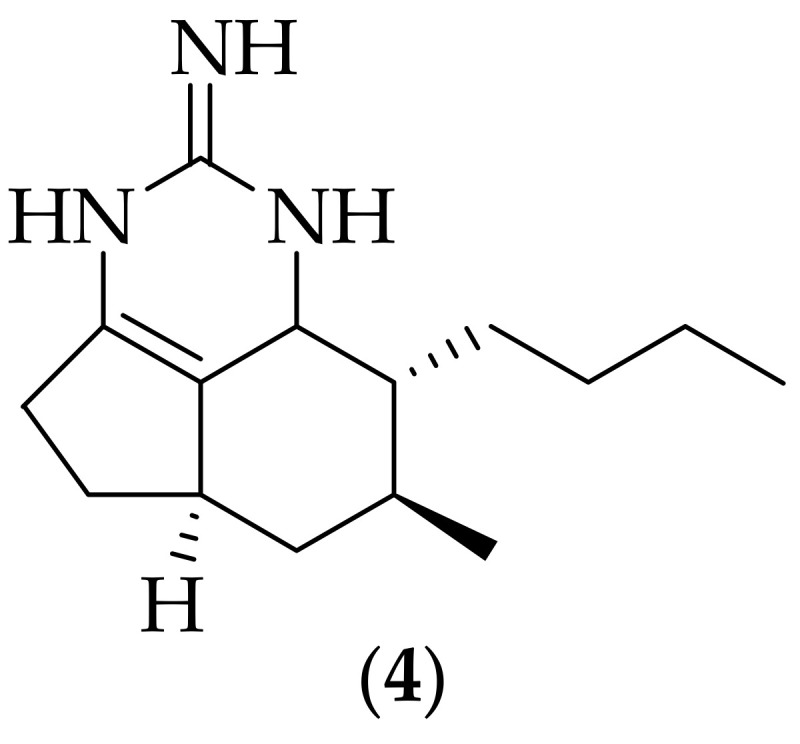
Structure of mirabilin B (**4**).

In 2004 Hua et al. published crystallographic and NMR studies on tricyclic guanidine alkaloids isolated from the sponge *Monanchora unguifera*, [12]. The authors reported that one of the isolated constituents of the methanol extract of *Monanchora unguifera* was mirabilin B, (**4**), Figure 2, (originally isolated from the marine sponge *Arenochalina mirabilis* [13]) which possessed antifungal activity against *Cryptococcus neoformans*, with an IC_50_ value of 7.0 μg/mL. The potency of this analog is still modest, and it would be valuable to learn its potency against other relevant fungal species, such as *Aspergillus fumigatus* and *Candida albicans*.

**Figure 3:**
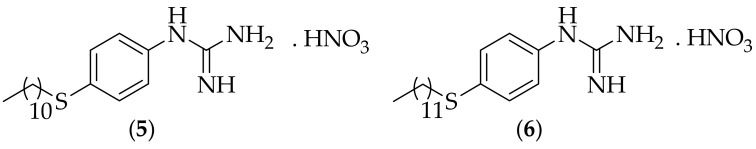
Phenyl guanidinium salt antifungal agents (**5**) and (**6**).

Furthermore, in 2004, the group of Braunerova and coworkers published their work on the synthesis and biological evaluation of structurally simple 4-substituted phenyl guanidinium salts, [14]. A total of twenty one analogs were synthesized, and from these, two molecules were highlighted, (**5**) and (**6**), Figure 3. Both compounds displayed comparable activity to the positive control, ketoconazole, against strains of *Aspergillus fumigatus* and *Aspergillus corymbifera*, and lower activity against six other fungal species. The group also reported that alternate salt forms (acetate and sorbate), and the free base of (**6**), were equipotent with the originally synthesized nitrate salt. It is encouraging that the two analogs described displayed good potencies, and additional work to expand the data on these derivatives would be welcome, along with the synthesis and evaluation of additional related compounds, such as incorporating additional substitution on the phenyl ring.

**Figure 4:**
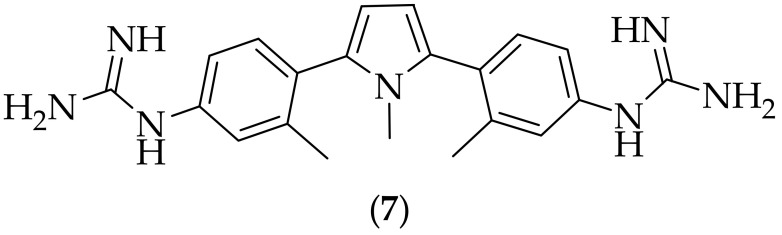
Pyrrole-diguanide antifungal derivative (**7**).

Having previously described antifungal analogs of azoles, [15], and tetrazoles, [16], Sinha et al. in 2005 reported on the antifungal activity of diguanidine-containing pyrrole-based derivatives, [17]. Eight analogs were prepared, and were compared in their antifungal activity to fluconazole and itraconazole. The compound of most interest to the group was (**7**), Figure 4, which was shown to possess MIC values of 1–8 μg/mL against seven *Candida* species, and an MIC of 16 μg/mL against *Aspergillus fumigatus*-these values were superior to fluconazole for five of the *Candida* species. The group also found that the position of the guanidine group on the phenyl ring was important for activity, with 2-, and 3-substituted guanidine derivatives showing lower antifungal activity. With the potencies observed for (**7**) being reasonable against certain *Candida* species, it would be interesting to have this compound evaluated in an *in vivo* experiment against candidiasis.

**Figure 5:**
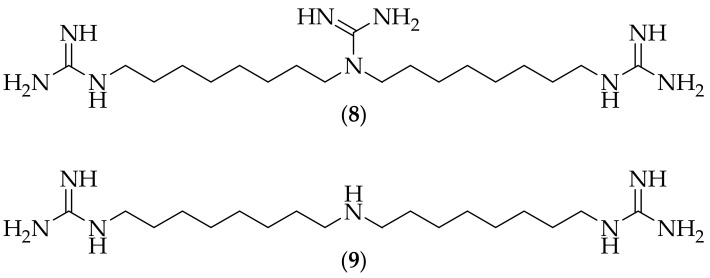
Guanidine-containing small molecules (**8**) and (**9**) separated from guazatine.

In 2007 Dreassi and coworkers reported on the analysis of guazatine and the antimycotic properties of the principal components, [18]. Guazatine is a non-systemic contact agricultural fungicide which comprises a mixture of reaction products from polyamines. The group carried out the identification and separation of guazatine by LCMS, and found that the most active antifungal components were the triguanide (**8**), and the bisguanide (**9**), Figure 5. The authors compared the activity of the isolated fractions of guazatine to fluconazole, and found that (**8**) and (**9**) were notably effective against fluconazole-resistant clinical isolates of *Candida albicans*, *Candida krusei*, and *Candida tropicalis*, with (**8**) having MIC_50_ values of 5 μM, 10 μM, 1.25 μM respectively. The efficient synthesis of (**9**) has been published, also in 2007 [19]. With (**8**) and (**9**) showing only modest *in vitro* potency against *Candida* species, the next step for these compounds would appear to be testing them against a broader range of molds and yeasts, to see if superior activtiy could be discovered.

**Figure 6:**
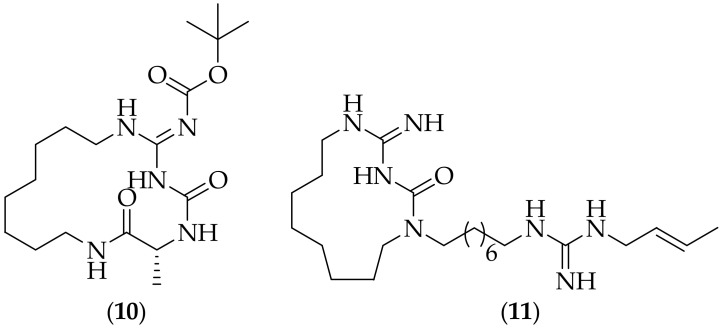
Cyclized derivatives (**10**) and (**11**) related to guazatine.

Two years after the initial report of the activity of the guazatine components, members of the same group reported on cyclized versions of some compounds related to guazatine components, [20]. An example of the compounds synthesized is compound (**10**), Figure 6, and the authors mention that the isolated compounds show “promising” biological results. The same group published further on guazatine-related derivatives in 2009, [21]. In this publication they reported novel cyclic analogs related to guazatine components, and the compound that the authors found most interesting was compound (**11**), Figure 6. This analog displayed good activity against the eleven *Candida* species tested against, with MIC values between 1.25 μM and 20 μM, but was less active against eight *Aspergillus* species, with MIC values between 4 μM and 73 μM. A patent on this work has also been published, [22], which includes structural information and *in vitro* antifungal activities for twelve analogs.

**Figure 7:**
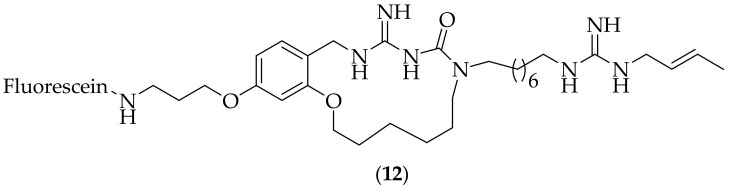
Fluorescent probe derivative (**12**) based on a cyclized analog of guazatine.

The Botta group has published an additional paper focused on compound (**11**), Figure 6 [23]. In this paper they describe both the *in vitro* characterization and *in vivo* efficacy of the compound. Compound (**11**) was tested against fourteen yeast strains, with time points at 24, 48, and 72 h, and the MIC values were determined to be between 5 and >80 μM. A transcriptional analysis of *Candida albicans* exposed to compound (**11**) was presented, which showed that at two time points, 15 and 45 min, there were 48 genes commonly up-regulated, and 27 genes commonly down-regulated. Two of the genes up-regulated were CDR1 and CDR2 (ATP binding cassette transporters), and the susceptibility of *Candida albicans* CDR1/CDR2 mutants when exposed to (**11**) was investigated. The results of these experiments showed that surprisingly the mutants became more resistant to (**11**), compared to the wild type strain. The authors hypothesized that there was a build-up of (**11**) in cells, and investigated this using fluorescence microscopy. Three fluorophore-containing macrocyclic analogs of (**11**) were synthesized, with the first two incorporating the fluorophore on the side-chain amine/guanidine moiety, and were inactive. The third fluorophore synthesized, (**12**) is shown in Figure 7, and this compound displayed low antifungal MIC activity of 80 μM. Compound (**12**) was used in a confocal microscopy examination of *Candida albicans* and *Cryptococcus neoformans*. The experiment showed that fluorescence was mostly associated with the cytoplasm, which confirmed intracellular accumulation. The observed fluorescence was found to be in a “patchy distribution” in some cells, suggesting that “the compound can interact with multiple, and/or differently located targets within the fungal cell”. Compound (**11**) was also tested in an *in vivo* experiment of invasive candidiasis in immunocompetent mice. Doses of 20 and 40 mg/kg were efficacious in reducing the fungal burden in kidney and spleen of mice infected with a fluconazole susceptible strain of *Candida albicans*. *In vivo* experiments were additionally carried out on (**11**) at 40 mg/kg against two other *Candida albicans* strains, one that is susceptible to azole drugs, and one that was azole resistant. The compound was effective in reducing the fungal burdens in target organs, kidney and spleen, for both strains. With (**11**) showing promising *in vivo* efficacy in mice, it would be highly valuable to assess its disease treating effectiveness in other species, starting with rats, and to generate *in vitro* absorption, distribution, metabolism, and excretion (ADME) data for the compound.

A follow-on paper was published by the same group in 2020, [24]. In this paper they performed additional evaluation of compound (**11**), which they named BM1. The compound was tested against sixty eight clinical isolate strains of six *Candida* species, and was shown to possess MIC ranges of 0.125–2 μg/mL for *Candida albicans* strains, 2 μg/mL for *Candida tropicalis* strains, and 8–64 μg/mL for *Candida parapsilosis*, *Candida glabrata*, *Candida krusei*, and *Candida auris* strains. BM1 was tested *in vivo* in rats, where it was found to have affinity for the renal system.

**Figure 8:**
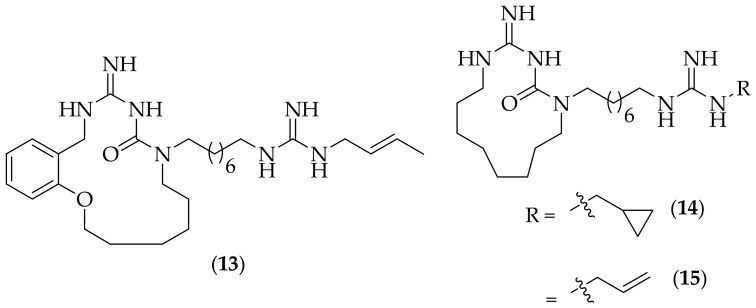
Cyclic guazatine-related analogs (**13**), (**14**), and (**15**).

The Botta group also published a paper in 2013 to expand upon their previous cyclic guatazine-related work , this time describing alterations in ring size, incorporation of polar moieties in to the macrocyclic ring, and guanidine alkyl group variations [25]. Seven analogs were synthesized, and three derivatives were highlighted, (**13**), (**14**), and (**15**), Figure 8. The three compounds showed comparable *Candida* MIC_90_ activity to the positive controls utilized in the various experiments, fluconazole, voriconazole, and amphotericin B, with activity against fluconazole-resistant *Candida* species, and amphotericin-resistant *Candida* strains. For this paper there is only MIC data presented for *Candida* species, and it would be informative for there to be a broader screen of other fungal species, such as *Fusarium solani* to ascertain the breadth of antifungal activity.

**Figure 9:**
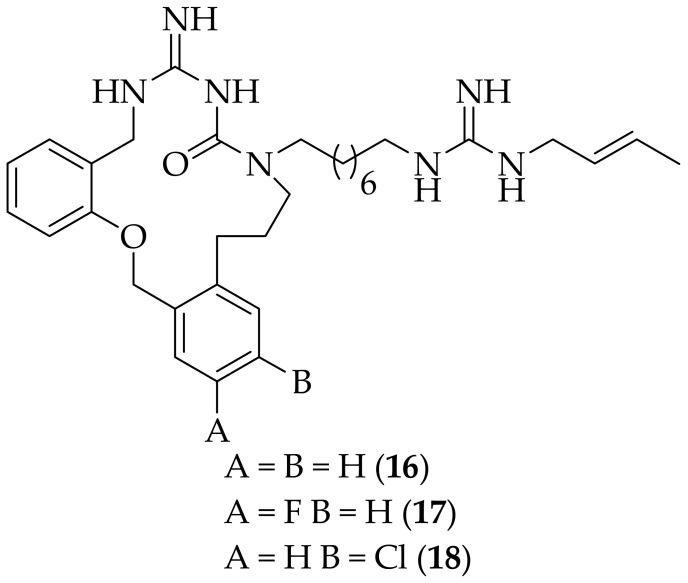
Arylated derivatives (**16**), (**17**), and (**18**) of cyclized guazatine-derived analog (**13**).

In 2022 novel derivatives of (**13**) were described by Dreassi et al. [26]. In this work an additional aromatic ring was incorporated in to the macrocycle of (**13**) so as to explore the chemical space of the macrocyclic core. Seven analogs were synthesized, and tested for their antifungal activity against one hundred strains of eight *Candida* species, and *Cryptococcus neoformans*. From these seven analogs, three were highlighted, (**16**), (**17**), and (**18**), Figure 9. Compound (**16**) had MIC_90_ values as low as 1 μg/mL, and compounds (**17**) and (**18**) both had MIC_90_ values as low as 1.56 μg/mL. The three compounds were investigated further, and were found to have low cytotoxicity in a FIBRO cell line (CC_50_ at 24 h of 38.5–0100 μg/mL), which were far higher than the MIC_90_ values. Parallel artificial membrane permeability assays (PAMPA) were carried out on the three derivatives, along with (**13**), and the results showed that there was no improvement in the permeability of the new analogs compared to (**13**), with all four compounds displaying membrane retention percentages of 51–55%. Clearly the aim of improving the membrane permeability was not achieved in this work, and it may be that the increase in molecular weight from (**13**) to (**16**), (**17**), and (**18**) is the problem, as it is known that permeability typically decreases as the molecular weight of compounds increase [27].

### 1.2. Guanidine-Containing Antifungal Agents 2008–2011

**Figure 10:**
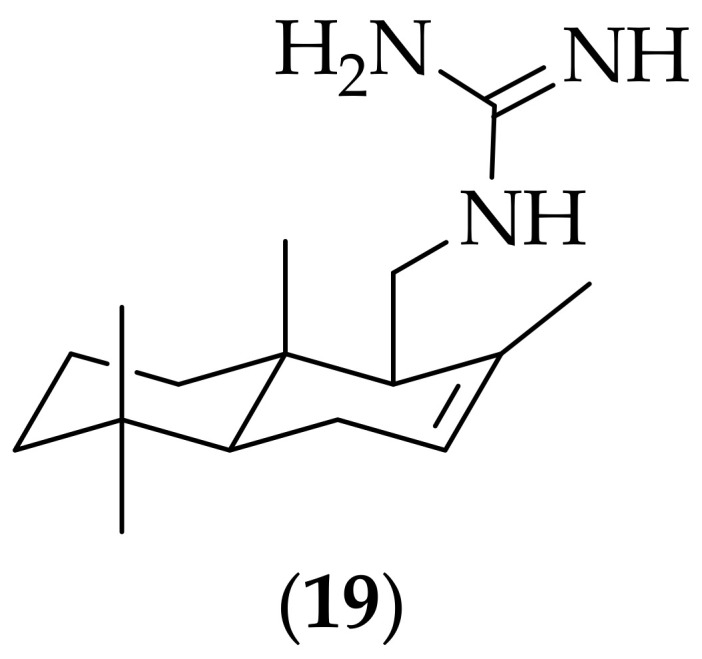
Drimenol-derived antifungal compound (**19**).

Zarraga and coworkers published their work in 2008 on a guanidine-substituted version of drimane, [28]. The drimane scaffold belongs to a family of natural sesquiterpenes, that can be isolated from marine and terrestrial sources, and which are known to have biological activity [29]. The analog that was synthesized in four steps starting from drimenol, was compound (**19**), Figure 10. (**19**) was shown to have an MIC value of 32 μg/mL against a strain of *Candida albicans*, which was found to be superior to the antifungal activity of the starting drimenol (MIC: 125 μg/mL). The potency of (**19**) is only modest against the strain of *Candida albicans* tested for, and it could be worthwhile to assess its potency versus a broad range of fungal species to see if higher potency can be found.

**Figure 11:**
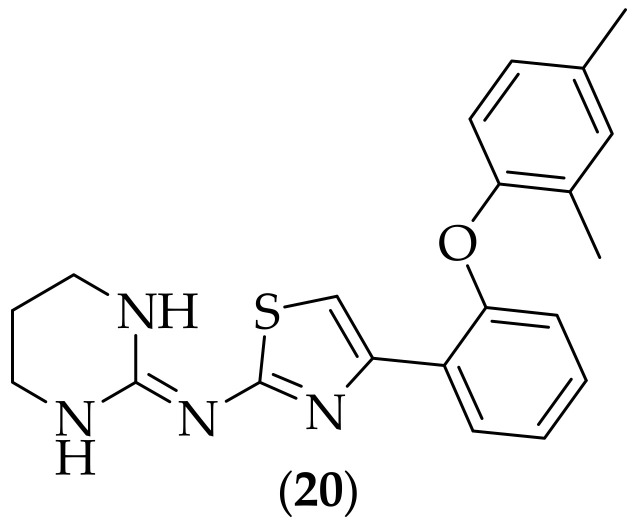
Structure of abafungin (**20**).

Furthermore, in 2008, Borelli and coworkers described investigations in to the antifungal activity and mechanism of action of abafungin (**20**), Figure 11, [30]. Abafungin was originally synthesized by Bayer AG, and was discovered when the company was screening for compounds related to famotidine, to act as antagonists for the H2-receptor. Abafungin was found to display potent antifungal activity against both yeasts (including *Candida* species), and molds (including *Aspergillus* species), with MIC values of between <0.06–8 μg/mL. The authors investigated the mechanism of action of (**20**), and found that one target was the inhibition of transmethylation at the C24 position of a sterol side chain of lanosterol, catalyzed by the enzyme sterol-C24-transmethyltransferase, and that a second mechanism of action appeared to be a direct effect on the fungal cell membrane. Abafungin has been marketed, as Abasol, by York Pharma as a topical cream formulation for dermatomycoses, [31]. The idea of repurposing agents for alternate targets continues to be a popular one, [32], and given the high potency found for abafungin against medically important fungal species such as *Aspergillus fumigatus*, along with the discernment of the mechanisms of action, it would have been informative for there to have also been *in vivo* experiments in disseminated infection models carried out with the compound, for example an aspergillosis model.

**Figure 12:**
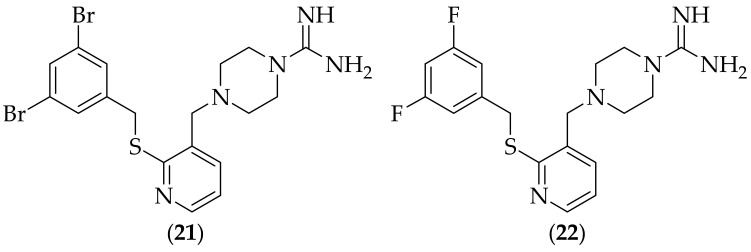
Fungistatic and fungicidal piperazine-1-carboxamidines (**21**) and (**22**).

Researchers published work in 2009, where they discovered guanidine analogs on a piperazine-based scaffold, [33]. A library of 500 compounds were screened for fungicidal activity through induction of reactive oxygen species (ROS) accumulation, and from this screen, the class of piperazine-1-carboxamidines emerged through two hits. Structure activity relationship (SAR) work was carried out on this series, with twenty four compounds being synthesized. There were two classes of compounds discovered; fungistatic, and fungicidal for *Candida albicans*. An exemplary compound from the fungicidal class is (**21**), Figure 12, which was found to accumulate reactive oxygen species, and to not be cytotoxic. A relatively close analog of (**21**), (**22**), was shown to be fungistatic. It is surprising that two such closely related analogs as (**21**) and (**22**) would generate different effects on *Candida albicans*, and raises questions as to the true nature and source of the antifungal effects. The involvement of reactive oxygen species in fungicidal activity was explored utilizing co-incubation with ascorbic acid, an antioxidant, which showed that there was antagonism between ascorbic acid and compound (**21**), and two other analogs. This result was interpreted as indicating the direct involvement of reactive oxygen species in the fungicidal activity of the compounds. The authors acknowledge that it is not clear from their research whether the build-up of reactive oxygen species is caused by enhanced reactive oxygen species production, or by the decreased ROS degradation. It is not clear why the reactive oxygen species generated by (**21**) and (**22**) would display specificity for fungal cells over mammalian cells, and this question should be investigated.

**Figure 13:**
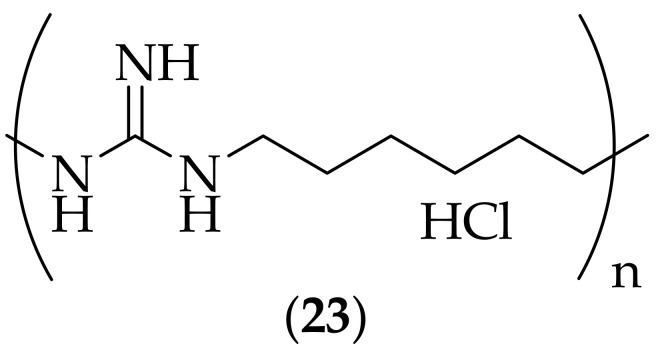
Structure of polyhexamethylene-guanidine hydrochloride (PHMGH) (**23**).

The antifungal activities of polyhexamethylene-guanidine hydrochloride (PHMGH), (**23**), Figure 13, were investigated by Koffi-Nevry et al. in 2011, [34]. The authors tested the agent against a range of thirty one fungal strains, including *Aspergillus* and *Mucor* species, and found that the MIC and MFC values ranged from <0.01–1.9 mg/mL, and noted that *Aspergillus* species were the least susceptible, with MFC values of 1.9 mg/mL. The compound has been used in South Korea as a disinfectant for household humidifiers. However, an outbreak of severe lung disease in the country between 2006 and 2011 was traced to the use of the compound, [35], and the product was banned. No additional cases were found once the ban had taken effect.

A separate research group carried out additional investigations on polyhexamethylene-guanidine hydrochloride (**23**), [36], with a view to testing the agent against a variety of pathogenic fungi, and elucidating its mechanism of action. The compound was found to possess antifungal activity comparable to, or superior to amphotericin B versus the five fungal species investigated (two *Candida* species, *Malassezia furfur*, *Trichosporon beigelii*, and *Trichophyton rubrum*), with MIC values of 1.25–2.5 μg/mL, and to not cause hemolytic activity, whilst amphotericin B did have this effect. The researchers utilized *Candida albicans* as the model fungal strain to investigate the compounds mechanism of action. After treatment with PHMGH, changes in the size and granularity of cells were observed by flow cytometric and microscopy methods. A membrane study using 1,6-diphenyl-1,3,5-hexatriene labeling showed a large loss of phospholipid area in the plasma membrane. Additional studies confirmed that PHMGH exerted its fungicidal effect through the formation of pores in the cell membrane. It would be interesting to have seen additional experiments related to the mechanism of action of PHMGH, to understand how the pores are formed in the cell membrane.

In 2020 Collares and coworkers described their investigation in to the use of polyhexamethylene guanidine hydrochloride (PHMGH) (**23**) for the inhibition of *Candida albicans* biofilm growth on dentures, [37]. Denture stomatitis and oral candidiasis are potential disease states associated with fungal biofilm growth on dentures, and the group investigated the use of PHMGH for their treatment. A 72 h–mature *Candida albicans* biofilm was first generated on denture liner specimens, and was treated with PHMGH. Solutions of PHMGH between 0.125 and 0.50 weight% in water were found to leave no detectable *Candida albicans* colonies after a ten minute immersion, and the 0.50 weight% solution was found to leave no detectable colonies after a five minute immersion.

In 2022 the group of Heaselgrave et al. published their research in to the use of PHMGH, polyaminopropylbiguanide (PAPB), and guazatine, in comparison to the contact lens disinfectant constituent polyhexamethylene biguanide (PHMB), for the treatment of ocular pathogens [38]. The researchers found that PHMGH was significantly more active (MIC: 0.5 μg/mL) than PHMB (MIC: 3.9 μg/mL) against a strain of *Candida albicans*. The four compounds were also tested over a 24 h period for their ability to reduce the viability of *Candida albicans* on contact lenses, and this experiment showed that PHMGH on average reduced the fungal viability by 4.05 log_10_ units at 24 h. The reduction in fungal viability by PHMGH is impressive, and suggests that additional work should be carried out in this area.

**Figure 14:**
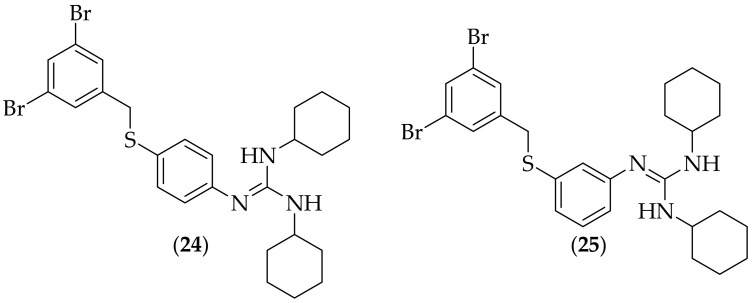
Fungicidal benzylsulfanyl-phenylguanidines (**24**) and (**25**).

In 2011 a group of researchers from Belgium and the Netherlands reported their work on fungicidal benzylsulfanyl-phenylguanidines, [39]. Following on from their work on piperazine-carboxamidines, [33], the group sought to make hybrid analogs of the piperazine-carboxamidines and abafungin (**20**), Figure 11, due to their perceived three-dimensional structural similarity. A total of twenty analogs were synthesized, and the compounds were evaluated for their minimum fungicidal concentration (MFC) against strains of *Candida albicans* and *Candida glabrata*. Five of the compounds showed MFC values of 5 μM for both fungal strains, and four of these were tested in a *Caenorhabditis elegans* (*C. elegans*) model of *Candida albicans* infection. Two of these compounds were shown to be efficacious in increasing survival *in vivo*, and they are shown in Figure 14. These two compounds were also evaluated for the ability to eradicate *Candida albicans* biofilms, and the biofilm eradicating concentration for (**24**) was 121.0 +/− 17.2 μM, and for (**25**) was 19.0 +/− 12.1 μM- both were shown to be superior in this respect to fluconazole. It is encouraging that (**25**) had a good biofilm eradicating concentration, however the large statistical variation in activity suggests that more careful experiments need to be carried out to ascertain a more accurate biofilm eradicating concentration. The biofilm eradicating concentration of (**25**) does not appear to be particularly impressive at ~19 μM

**Figure 15:**
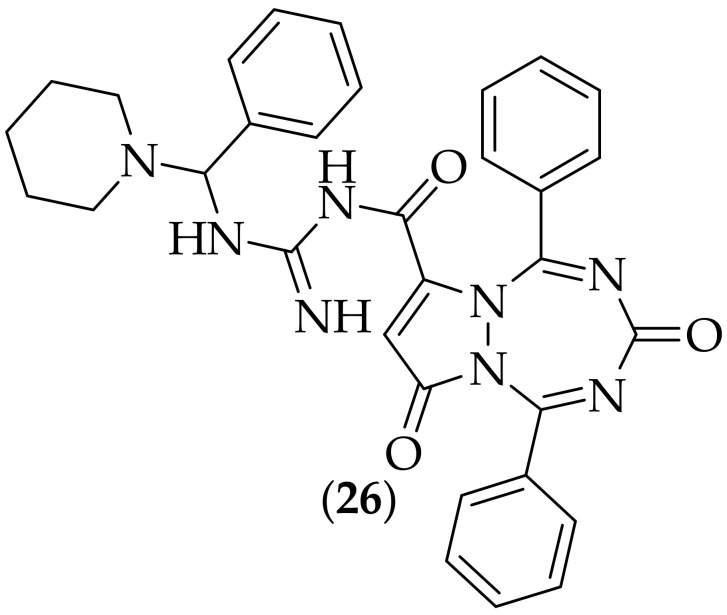
Mannich base antifungal derivative (**26**).

Furthermore, in 2011, Modi and coworkers described their research in to Mannich bases of a pyrazolo [1,2-A][1.2.4.6] tetrazepine-3,7-dione ring system, [40]. The guanidine analog synthesized was (**26**), Figure 15. This compound was tested in a filter paper disk method of evaluating the zone of inhibition for *Candida albicans*, for which at a concentration of 1000 μg/mL a result of 8 mm was found, which was similar to the result found for fluconazole at 250 μg/mL of 15 mm. The activity of (**26**) is clearly modest, and distinct improvements in potency would be required for this type of analog to become more interesting as an antifungal agent.

### 1.3. Guanidine-Containing Antifungal Agents 2012–2016

**Figure 16:**
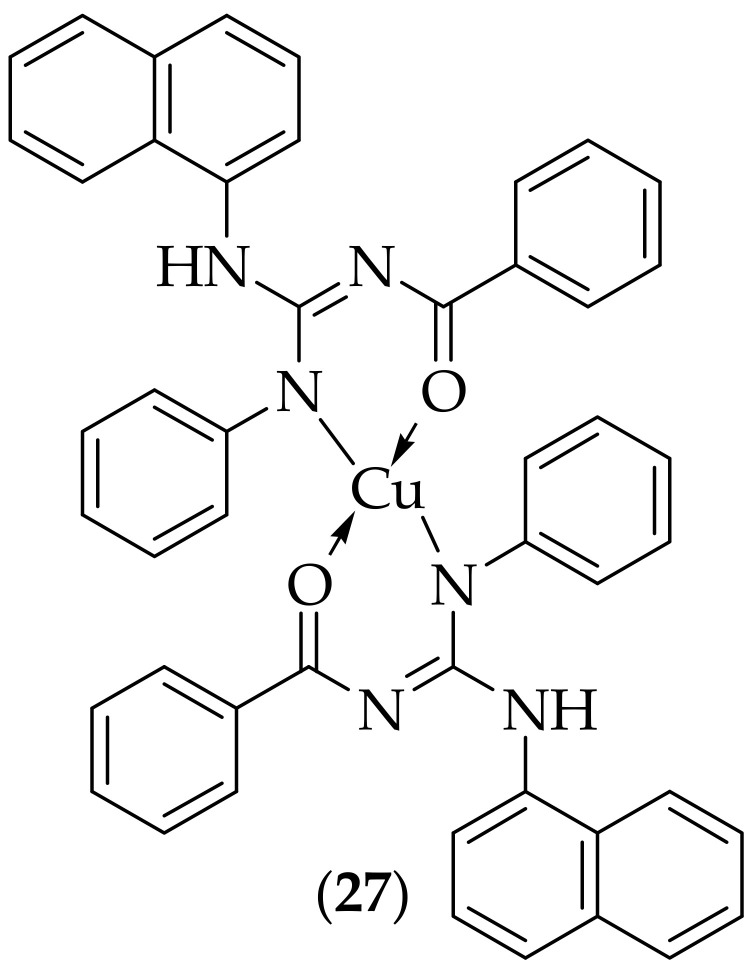
Copper (II) complex antifungal agent (**27**).

A series of guanidine ligands, and the homoleptic copper (II)) complexes thereof were evaluated for their antifungal activity by Badshah et al. in 2012, [41]. Eight copper (II) complexes were tested for antifungal activity using an Agar tube dilution method, and the results showed that the most active compound was (**27**), Figure 16, which displayed good potency against *Mucor*, *Aspergillus niger*, and *Fusarium solani* strains, with activities of 53–76% inhibition. The non-complexed ligand of (**27**) showed lower activity against these strains (37–61% inhibition), indicating that the complexation was having a slightly beneficial effect on the antifungal activity. An LD_50_ of 779 μg/mL was found for (**27**) in brine shrimp. It is not clear that these complexes have a path forward towards greater activity and efficacy *in vivo*.

**Figure 17:**
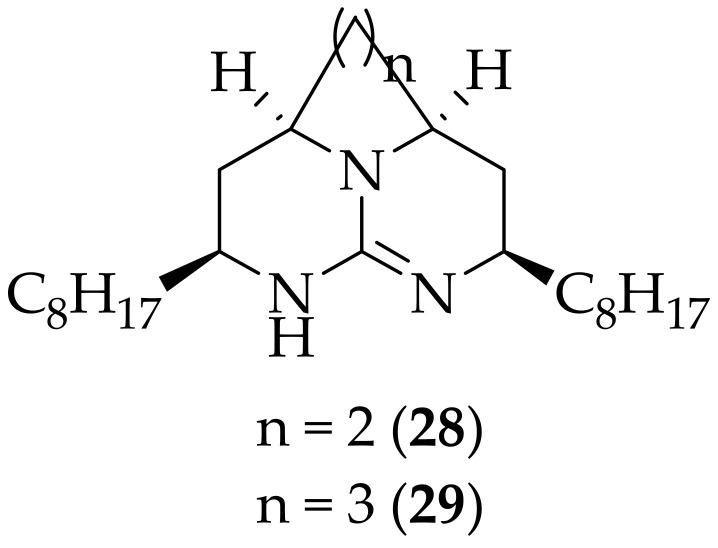
Antifungal derivatives (**28**) and (**29**) of batzelladine K.

Tricyclic guanidine analogs of batzelladine K, a polycyclic marine alkaloid, were synthesized and evaluated for their antifungal activity by Bhutani and coworkers in 2013, [42]. Fifty derivatives were made, and the authors found that twenty two of the analogs possessed some antifungal activity. The scientists focused on (**28**) and (**29**), Figure 17, as being of particular interest due to their higher antifungal potency. Compounds (**28**) and (**29**) showed IC_50_, MIC, and MFC values between one and ten-fold higher than those found for the positive control amphotericin B, versus three *Candida* strains, *Cryptococcus neoformans*, and *Aspergillus fumigatus*. Analogs (**28**) and (**29**) display good activity, particularly against *Candida krusei*, and *Cryptocossus neoformans* (~3 μM) and it would seem that the next step with these derivatives would be to test them in *in vivo* experiments against candidiasis and/or cryptococcosis.

**Figure 18:**
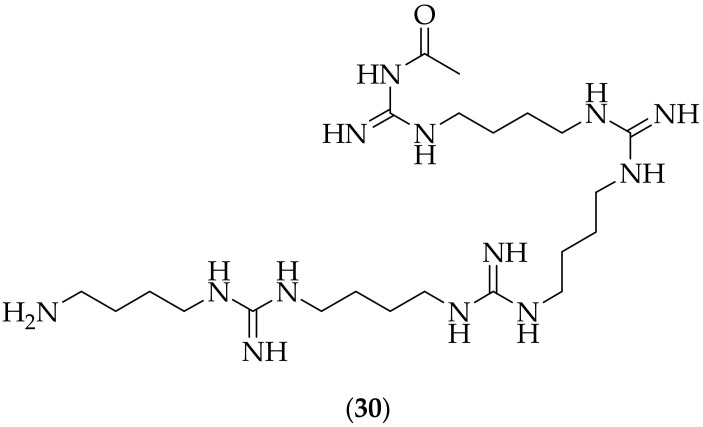
Structure of cabanillasin (**30**).

A metabolite isolated from *Xenorhabdus cabanillasii*, named cabanillasin (**30**), Figure 18, was described by Gualtieri et al. in 2013 [43]. Cabanillasin was tested for its antifungal activity against yeasts and filamentous fungi involved in nosocomial infections. The strains were tested over both 24 and 48 h, with the results showing that the activities at 48 h were lower than at 24 h, for which the explanation was the potential chemical instability of cabanillasin. It would be informative to have cabanillasin tested for its chemical stability, so as to be able to determine if this is the reason for the lower activity at 48 h, or if there is some alternate explanation. At 24 h, (**30**) was shown to be highly active against strains of *Candida albicans* (IC_50_: 0.78 μg/mL), *Candida lusitaniae* (IC_50_: 1.56 μg/mL), *Candida glabrata* (IC_50_: 6.25 μg/mL), and *Candida krusei* (IC_50_: 6.25 μg/mL). At a 48 h timepoint, cabanillasin was found to have only low activity against the filamentous fungi *Aspergillus fumigatus* and *Rhizopus oryzae*. Cytotoxicity testing revealed that the LD_50_ of (**30**) in human prostatic carcinoma cells (PC-3) was 47 μg/mL, whilst in human normal mammary epithelial cells (hTERT HME-1) the LD_50_ was 25 μg/mL. With cabanillasin showing good *in vitro* potency and lower cytotoxicity, it would be valuable to have the compound tested *in vivo*, for example in a *Candida albicans* experiment.

**Figure 19:**
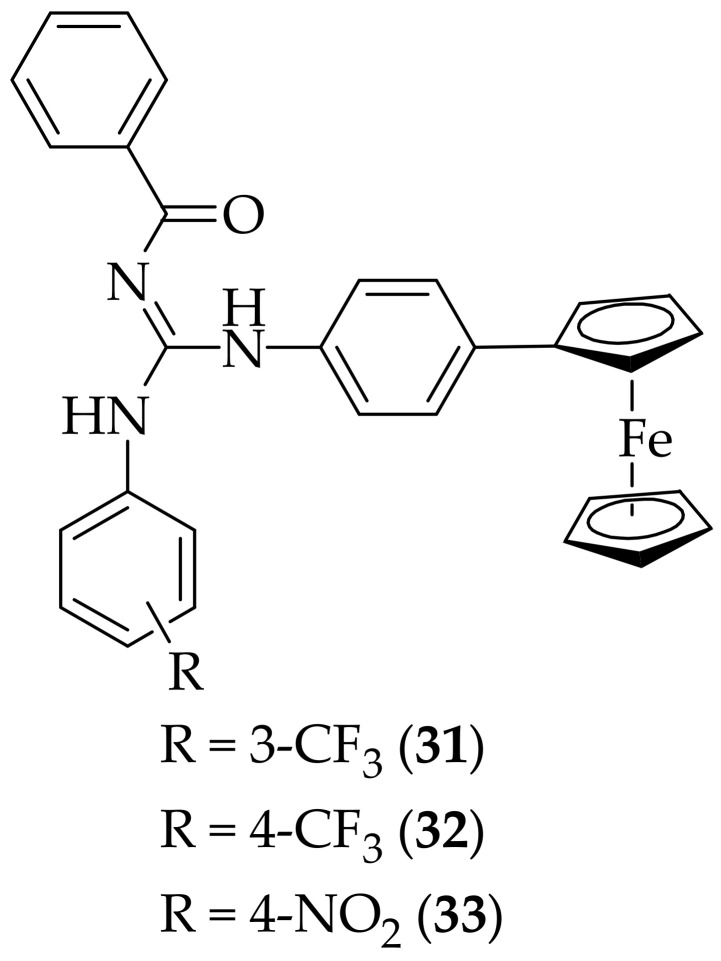
Ferrocene-based guanidine-containing antifungal agents (**31**), (**32**), and (**33**).

Following on from their previously described work on the copper (II) complexes of guanidine-containing ligands [41], ferrocene-based guanidine-containing complexes as antifungal agents were published by Badshah and coworkers, in 2014 [44]. Eight analogs were tested for the area of fungal growth, and zone of inhibition, for strains of *Fusarium moniliforme*, *Aspergillus fumigatus*, and *Aspergillus flavus*. Three of the compounds, Figure 19, were found to produce positive results- results which were comparable with, or slightly less effective than the positive control, terbinafine- with zones of inhibition for (**31**), (**32**), and (**33**) of 65–96%. The other five complexes were found to be appreciably less effective. The authors speculate that the presence of electron-withdrawing groups on the aryl ring, as seen in (**31**), (**32**), and (**33**), causes a decrease in the basicity of the guanidine, and an increase in the lipophilicity of the compounds, which is beneficial to antifungal activity. It is noteworthy that the three analogs shown in Figure 19 have similar potency to terbinafine, and suggests that perhaps additional analogs containing more electron deficient aromatic rings ought to be synthesized, to see if the authors’ hypothesis is correct.

**Figure 20:**
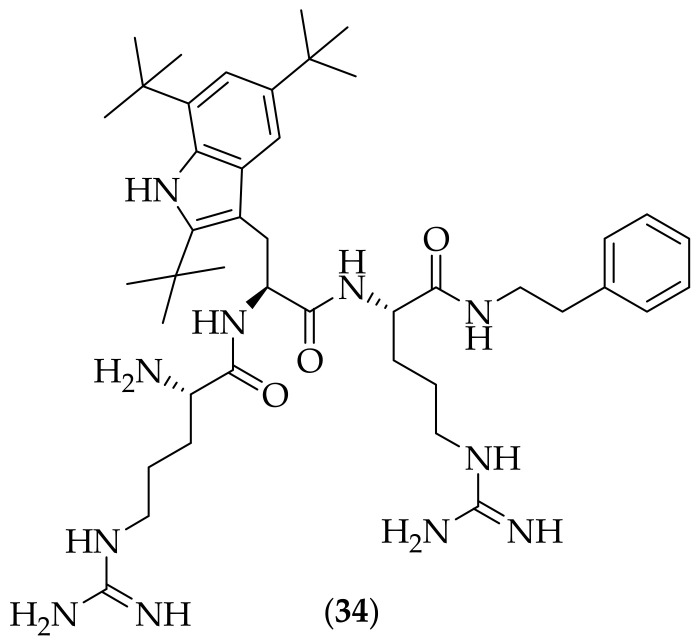
Short cationic antimicrobial peptide (LTX-109, AMC-109) (**34**).

In 2016, Svenson et al. reported their studies on short cationic antimicrobial peptides, [45]. The group synthesized five antimicrobial peptides, designed to illustrate diversity in the central amino acid as well as variation in the C-terminal functional group- all five peptides contained two guanidine groups. The five peptides were tested against twenty four pathogenic strains of fungi. Of the five polypeptides, three were found to possess generally potent antifungal activity, and the compound that was focused upon was (**34**), Figure 20, with MIC values of between 4 and 64 μg/mL, although these potencies were appreciably lower than for the positive control utilized, amphotericin B [45]. This compound has previously been selected as an antibacterial clinical development candidate by Lytix Biopharma, which they named LTX-109 [46]. Work with this compound is continuing with Amicoat (with the compound renamed as AMC-109) [47] and Pharma Holdings [48]. Compound (**34**) was tested for its EC_50_ against human erythrocytes, which was found to be 175 μg/mL. The performance of compound (**34**) was analyzed in specific candidiasis and onychomycosis studies. In the candidiasis study, the compound was compared to terbinafine in an assay utilizing human skin. A 2% hydrogel formulation of (**34**) produced significant reduction in ATP levels against *Candida albicans* compared to untreated references. The compound also showed a significant increase in treatment efficacy compared to terbinafine. In the onychomycosis study, (**34**) was tested versus amorolfine as positive control and deionized water as placebo. The study, run over 48 h, demonstrated that compound (**34**) lowered recovery of ATP from nail samples, compared to the infection control and placebo. Four nail penetration enhancers were also investigated in conjunction with (**34**), and it was found that MedNail was the most effective, producing baseline recovery of ATP comparable to the non-infected controls. Given that (**34**) was selected as a development candidate by Lytix Biopharma, and is being investigated by Amicoat and Pharma Holdings, it would be expected that the compound has a good overall profile, and with its efficacy in both candidiasis and onychomycosis experiments, the compound is highly interesting.

**Figure 21:**
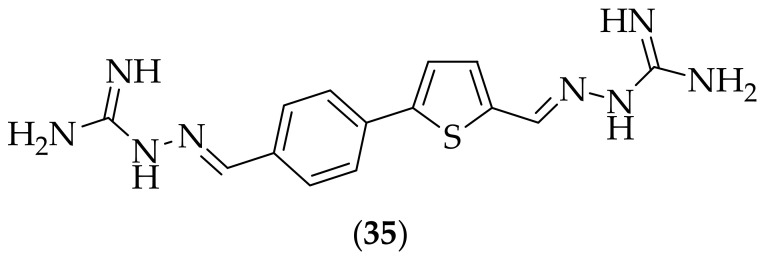
Lead thiophene-based bis-guanylhydrazone antifungal compound (**35**).

In 2016 Opsenica and coworkers described thiophene-based guanylhydrazones as antifungal agents, [49]. The group synthesized ten guanylhydrazones, and tested them against eight fungal strains. The experiment showed that seven of the derivatives showed appreciable antifungal activity, with the compounds containing two guanidine moieties generally displaying higher antifungal potency. The compound that was selected for further study, due to its higher antifungal activity (MIC values from 0.50 – >250 μg/mL) and lower cytotoxicity value (10 μg/mL), was (**35**), Figure 21. Compound (**35**) was evaluated in a time-kill experiment, which showed that at 12 h of incubation there was a pronounced fungistatic effect on *Candida albicans*. The derivative was next tested for its effect on *Candida albicans* membranes using a concavaline/propidium iodide (PI)/4′,6-diamidino-2-phenylindole (DAPI) cell staining assay. The result of the assay showed that upon treatment with (**35**), membrane damage and cell death were observed in 20% of the cells, and that the treatment did not induce apoptosis. Compound (**35**) was also shown to disperse preformed *Candida* biofilms. The site of action of (**35**) was interrogated by the effect of the compound on sheep red blood cells, and it was determined that the compound did not act as a membrane disruptor as its primary mechanism. *In vivo* embryotoxicity was determined using zebrafish, which showed that the compound was less embryotoxic and teratogenic than the azole drug voriconazole. Overall compound (**35**) looks to be an interesting antifungal agent. The information that the compound is not a membrane disruptor is useful, but it would be more valuable to be able to understand how the compound exerts its effect.

**Figure 22:**
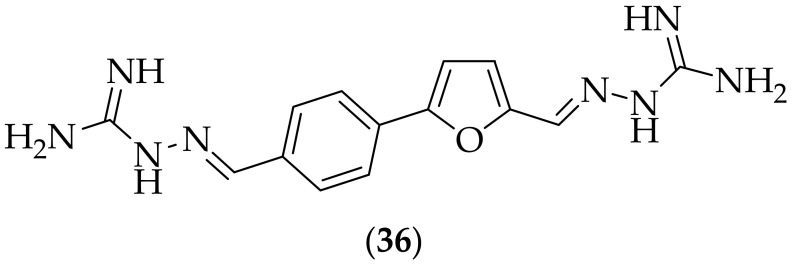
Furan-based bis-guanylhydrazone antifungal derivative (**36**).

In 2018 the same group published on additional bis-guanylhydrazones as effective agents for the treatment of candida infections, [50]. The group synthesized three new bis-guanylhydrazones, as close analogs of their previous lead molecule (**35**), and tested them against four *Candida* strains. The results showed that compound (**36**), Figure 22, was the most active analog, with MIC values comparable to the two positive controls, nystatin and amphotericin B, and that (**36**) showed the lowest cytotoxicity against healthy human fibroblasts (MRC5) of the three analogs (40 μg/mL). The potential of compound (**36**) to interact with chromosomal DNA from *Candida albicans* was investigated, with the result showing that the compound did appear to interact with DNA, for which additional evidence was presented though the use of circular dichroism spectral analysis, which showed that the compound significantly perturbs the conformation of double stranded template DNA (dsDNA). The DNA-specific probe DAPI was utilized to investigate the pro-apoptotic effect of (**36**). The result of this work showed that there was a significant decrease in the intensity of the blue color associated with the DNA-DAPI complex in the *Candida albicans* cells treated with the MIC of compound (**36**), indicating perturbation of the conformation of the dsDNA. The reactive oxygen species (ROS) generation and accumulation caused by (**36**) was found to be significantly lower than for amphotericin B. The synergism between (**36**) and known antifungal agents was investigated, which showed that (**36**) and amphotericin B acted synergistically against *Candida albicans* and *Candida parapsilosis*, but that (**36**) did not act synergistically with nystatin or itraconazole against these two *Candida* species. As only three additional derivatives were synthesized this only provides a small sample size to select from. It would be instructive to see more analogs made, preferably of (**36**) as it is the superior compound, to incorporate substituents on to the phenyl ring, incorporate substituents on to the furan ring, phenyl replacements such as pyridyl and thiophene, and furan replacements such as oxazole and oxadiazole.

**Figure 23:**
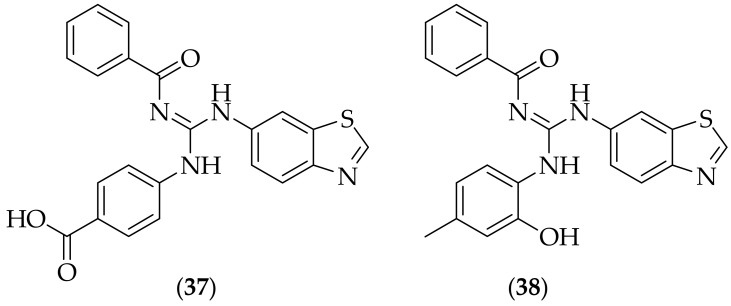
Lead guanidine-containing benzothiazole derivatives (**37**) and (**38**).

In 2016 a set of thirteen guanidine -containing benzothiazole analogs were synthesized and tested by Bhat and Belagali, [51]. The compounds were tested at 200 μg/mL for their zone of inhibition of *Candida albicans* and *Aspergillus niger*, and the two most interesting compounds were (**37**) and (**38**), Figure 23. (**37**) and (**38**) produced zones of inhibition for *Candida albicans* of 40 and 41 mm, respectively, and for *Aspergillus niger* the zones of inhibition were lower, 21 and 25 mm respectively. The MIC values for *Candida albicans* and *Aspergillus niger* were also calculated- for *Candida albicans* the MIC values were 3.125 μg/mL for (**37**) and 1.56 μg/mL for (**38**), and for *Aspergillus niger* the MIC values were lower at 12.5 μg/mL for both (**37**) and (**38**). The MIC values calculated for *Candida albicans* and *Aspergillus niger* for (**37**) and (**38**) are modest, and suggest the need for additional SAR investigation- changes such as additonal SAR on the aniline phenyl ring, substitution on to the benzoyl group, replacement of the benzoyl group, and replacement of the benzothiazole all appear to be reasonable approaches.

### 1.4. Guanidine-Containing Antifungal Agents 2017–2022

**Figure 24:**
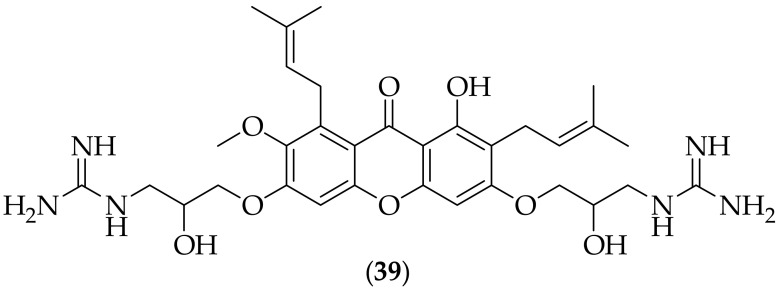
α-Mangostin derived antifungal analog (**39**).

Semisynthetic analogs of α-mangostin were described as possessing antifungal activity by Liu et al. in 2017 [52]. The group chose to synthesize derivatives of α-mangostin as it was known to possess modest antifungal activity, [53,54], and the scientists wished to incorporate cationic amphiphilic groups in to the molecule, so as to make the compounds membrane targeting, as it is known that fungal cell membranes are negatively charged [55,56,57], whilst mammalian cell membranes are neutral [57]. There were thirty one analogs synthesized, starting from α-mangostin, and one of the two compounds focused upon was (**39**), Figure 24. This compound was determined to have MIC values of 0.78 μg/mL for two strains of *Candida albicans*. The concentration of (**39**) needed to lyse 50% of rabbit red blood cells (HC_50_) was 48 μg/mL, showing selectivity for the fungal species. Compound (**39**) was further tested against four strains of *Candida albicans*, four strains of *Fusarium solani*, and five *Aspergillus* species. The results showed that (**39**) had MIC values of 0.78 μg/mL for the *Candida albicans* strains, 3.13 μg/mL for the *Fusarium solani* strains, and 3.13–6.25 μg/mL for the *Aspergillus* species. Compound (**39**) was tested in combination with six known antifungal agents, and there was synergy observed with only one, terbinafine. A time-kill kinetics experiment was carried out for (**39**), which showed that the compound had a rapid fungicidal effect on a strain of *Candida albicans*. The cytotoxicity of (**39**) was evaluated, and it was found that the IC_50_ was 64.1 μg/mL in human corneal fibroblasts. The mechanism of action of (**39**) was assessed using the DNA-binding dye Sytox Green, which readily penetrates compromised membranes of dead cells, but cannot cross the intact membranes of living cells. This experiment suggested that compound (**39**) was killing fungi by disrupting the fungal cell membrane. Finally, an *in vivo* experiment was carried out with (**39**), in a murine fungal keratitis experiment with a *Candida albicans* strain. The compound was dosed as a 0.2% solution, and was given over a six hour period. Relative to untreated controls, compound (**39**) produced a 93% reduction in fungal burden in the eye. With excellent MIC values against *Candida albicans*, synergism with terbinafine, low cytotoxicity, an implied mechanism of action, and *in vivo* efficacy, compound (**39**) has one of the most complete profiles of the antifungal agents presented. It would be interesting to have investigated if the derivative could also prove efficacious in other *in vivo* models, such as disseminated candidiasis.

**Figure 25:**
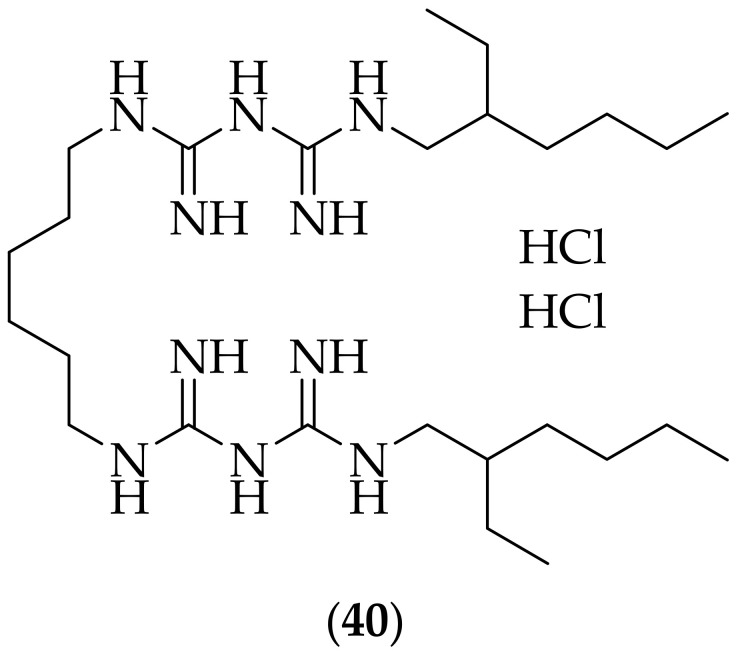
Structure of alexidine dihydrochloride (**40**).

In 2018, Uppuluri and coworkers published their research on the antifungal effects of the marketed anticancer/antimicrobial agent alexidine dihydrochloride (**40**), Figure 25, [58]. After screening a collection of 1200 FDA-approved off-patent drugs against strains of *Candida albicans*, *Candida auris*, and *Aspergillus fumigatus*, the group found that the most active agent was alexidine dihydrochloride. The group also tested the top six compounds from the original screening for their ability to inhibit biofilm formation, and showed that only two compounds, alexidine dihydrochloride and the organomercury agent thimerosal [59] (used as a vaccine preservative) were able to significantly kill 80% of mature biofilms (at <10 μM) of the same three fungal strains utilized in the initial screening. Dose–response investigations were carried out with (**40**) versus a range of fungal strains for three different growth conditions- planktonic, biofilm inhibitory, and mature biofilm effects. The compound showed good MIC (50% inhibition) values for almost all of the strains tested against, with MIC values of 0.15–20 μg/mL. Alexidine dihydrochloride was additionally shown to kill 50% of human umbilical vascular endothelial cells (HUVECS) and lung epithelial cells at concentrations 5–10-fold higher than the MIC needed to kill planktonically growing fungal pathogens. A synergistic effect of (**40**) with fluconazole was also demonstrated on *Candida albicans* biofilms, with a fractional inhibitory concentration (FIC) index (defined as the EC_50_ of the drug in combination/EC_50_ of the drug alone) of 0.42. Another drug repurposing effort yields positive results, with, in some cases, high *in vitro* potency, and synergism with a known antifungal agent. Studies to determine *in vivo* efficacy would be welcome to follow up upon this interesting discovery.

**Figure 26:**
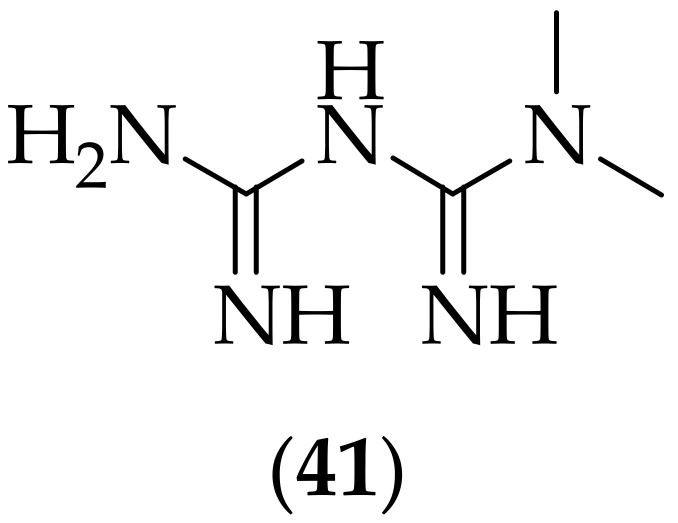
Structure of metformin (**41**).

Another guanidine containing drug, metformin (**41**) [60], Figure 26, an anti-diabetic medication, was shown in 2018 to enhance the antifungal activity of known antifungal agents against *Candida glabrata*, [61]. Metformin, along with related compounds phenformin, and buformin, were found to have MIC_50_ values against *Candida glabrata* of 9.34, 2.09, and 1.87 mg/mL respectively. Metformin itself was shown to have MIC values of between 9 and >25 mg/mL versus six *Candida* species. The ability of metformin to enhance the activity of various known antifungal drugs against a strain of *Candida glabrata* was investigated, with the results showing that the antifungal activity of voriconazole, fluconazole, and amphotericin B were all positively impacted by co-treatment with metformin, whereas micafungin was not positively impacted by co-treatment with metformin. Finally, (**41**) was found to decrease the MIC values of antifungal agents against drug-resistant strains of *Candida glabrata*. Seven resistant strains were investigated- for the four azole-resistant strains, voriconazole demonstrated a lowered MIC_50_ in combination with metformin, and fluconazole demonstrated a lowered MIC_50_ in combination with metformin. All micafungin resistant strains did not display any positive effect of micafungin in the presence of metformin, consistent with the earlier result. While the MIC_50_ values for metformin were not that high, it is interesting that the agent was able to display lowering of MIC_50_ in combination with voriconazole and fluconazole, and suggests that *in vivo* investigations are warranted with metformin in combination with azole antifungal agents, for the treatment of candidiasis.

**Figure 27:**
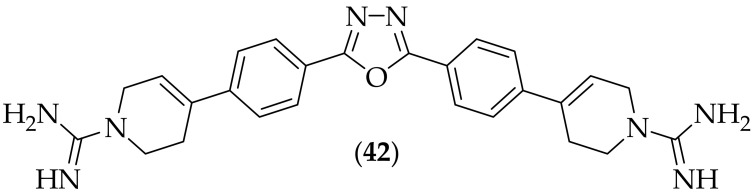
Bis-guanidine containing oxadiazole analog (**42**).

Our group have continued research upon small molecule host-defense peptide mimetics (smHDPM) as antifungal agents [62,63], aiming to recapitulate the effects of host defense peptides, but with a small molecule. Our published paper in 2019, [64], described five different compounds from our labs, which were tested for their antibacterial and antifungal activity. Of the five analogs, the bis-guanidine containing oxadiazole analog, (**42**), Figure 27, was of most interest due to its antifungal potency. Compound (**42**) bears some similarity to compound (**7**), which was published by Sinha et al. [18]. (**42**) was shown to have potent activity against strains of *Candida albicans*, *Aspergillus fumigatus*, and *Aspergillus flavus* (all MIC: 0.39 μg/mL. This compound was also found to have low CC_50_ values of 124 μM (3T3 cells), and 151 μM (HepG2 cells).

**Figure 28:**
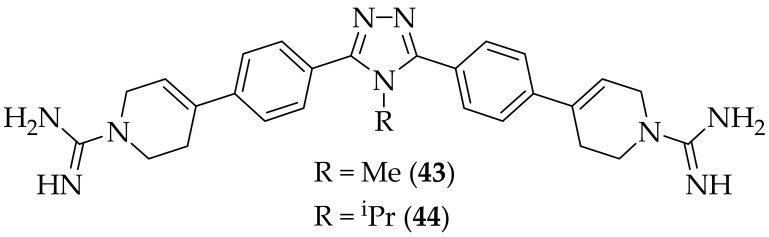
1,2,4-triazole bis-guanidine-containing antifungal derivatives (**43**) and (**44**).

Work continued in our group on smHDPM’s, and some additional results were published in 2021, [65]. Following on from the discovery of oxadiazole (**42**) [56], we investigated substitution on to the oxadiazole substructure, and the variation of the core of the molecule, It was discerned that the 1,2,4-triazole (**43**), Figure 28, was the optimal for antifungal potency of the three cores investigated. Focusing on the 1,2,4-triazole core, four different connectors between the phenyl ring and the guanidine were described, with the 1,2,5,6-tetrahydropyridine, found in (**42**), and (**43**), being the most active. Alterations to the alkyl substituent on the triazole nitrogen were also examined, and the compound that was focused upon was the N-isopropyl analog, (**44**). (**44**) was found to be highly potent, displaying MIC values of <1 μg/mL against four of the six fungal species tested against (*Aspergillus flavus*, *Aspergillus fumigatus*, *Fusarium falciforme*, and *Fusarium solani)*, along with MIC values of 0.2 μg/mL for *Mucor circinelloides*, and 0.78 μg/mL for *Mucor ramosissimus*, with these potencies being equal to, or superior to the comparator agents posaconazole and amphotericin B. Cytotoxicity evaluation showed that (**44**) had low CC_50_ values of 297 μM and 751 μM in 3T3 and HepG2 cells respectively. (**44**) was additionally found to possess excellent *in vitro* ADME properties. A patent on these systems has also been published, [66], which provides information on the over eighty analogs synthesized in this area.

**Figure 29:**
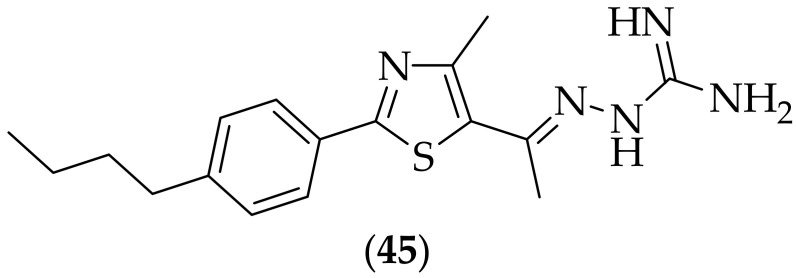
Lead thiazole-aminoguanidine analog (**45**).

Furthermore, in 2019, Seleem et al. described their work identifying a small molecule with both antifungal and antibiofilm properties, [67]. A library of eighty five thiazole-containing analogs, including thirty four derivatives that included a guanidine moiety, were tested for their MIC values against a strain of *Candida albicans*, and the most active compound was (**45**), Figure 29, which had an MIC of 0.5 μg/mL. Compound (**45**) was next tested against five strains of *Candida albicans* (MIC: 0.5–2 μg/mL), eight strains of *Candida auris* (MIC: 2 μg/mL), two strains of *Candida glabrata* (MIC: 0.25–1 μg/mL), one strain of *Candida parapsilosis* (MIC: 0.5 μg/mL), two strains of *Candida tropicalis* (MIC: 1 μg/mL), two strains of *Cryptococcus gattii* (MIC: 0.5 μg/mL), one strain of *Cryptococcus neoformans* (MIC: 0.5 μg/mL), and three strains of *Aspergillus fumigatus* (MIC: 2–4 μg/mL), and these values were found to be comparable to or superior to the two positive controls examined, amphotericin B and fluconazole. The ability of (**45**) to inhibit biofilms of *Candida albicans* and *Candida auris* was investigated, and it was shown that the compound was more effective at reducing biofilm formation by *Candida albicans*. The compound was was additionally tested for its ability to disrupt adherent *Candida albicans* biofilms, and it was found that at 4 x MIC, the compound reduced the metabolic activity of cells present in the biofilm by 66.3%. A kidney epithelial cell line (Vero) was utilized to evaluated the toxicity of (**45**), and it was shown that at concentrations up to 16 μg/mL, during a 24 h experiment, the cells appeared to be unaffected. The compound was evaluated for safety in *C. elegans*, where it was found that for the group of nematodes that were exposed to 20 μg/mL, 97% of worms survived over a four day experiment. (**45**) was finally tested in immunocompromised *C. elegans in vivo* efficacy experiments. In a *Candida albicans in vivo* experiment, 5 μg/mL treatment of (**45**) demonstrated > 70% survival of nematodes after four days, comparable with the positive control 5-Fluorocytosine at the same dose. In a *Candida auris in vivo* experiment, almost 70% of worms survived after four days following a 10 μg/mL treatment, which was found to be similar to 5-fluorocytosine. Thiazole derivative (**45**) shows excellent *in vitro* potency, with broad activity, and it would be instructive to have the agent tested in *in vivo* studies for diseases such as aspergillosis.

**Figure 30:**
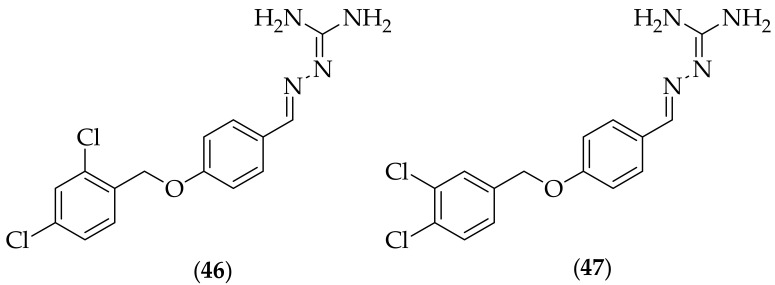
Aminoguanidine-containing antifungal compounds (**46**) and (**47**).

In 2020 Miyazaki et al. published their investigations in to novel antifungal agents, starting with a screen of 9600 compounds, [68]. Of the three hit compounds, one compound contained an aminoguanidine, (**46**), Figure 30, and sixteen derivatives of this compound were synthesized. The most active analog was the closely related derivative (**47**). Compound (**47**) displayed activity against six *Candida* species, *Cryptocccus neoformans*, three *Aspergillus* species, and *Rhizopus oryzae*, with IC_50_ values between 12.5 and 50 μM (4–17 μg/mL). Compounds containing modified guanidine groups were appreciably less active. (**47**) was evaluated for its IC_50_ against a known multi-drug resistant *Candida albicans* strain, NCPF8985, and was shown to possess superior activity to six known antifungal drugs, with only amphotericin B being more active. (**47**) was also shown to have anti-biofilm activity against *Candida albicans*, although the activities were lower than for fluconazole (planktonic) and amphotericin B (planktonic and sessile). The cellular cytotoxicity in human A549 cells of (**47**) was determined to be 8 μg/mL, which is in the same range as the antifungal activities observed, which raises the question of whether the antifungal activity observed with (**47**) was due in part to cytotoxicity. Additional studies should be carried out to determine if the activity of (**47**) is caused by cytotoxicity, and if not, the compounds mechanism of action.

**Figure 31:**
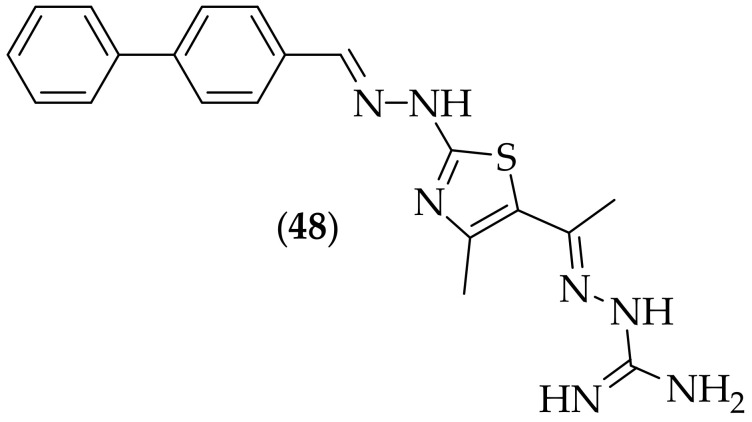
Guanidine-containing GlcN-6-P synthase inhibitor (**48**).

Furthermore, in 2020, Ahmed and coworkers reported their investigations in to inhibitors of GlcN-6-P synthase, which catalyzes the first step in the biosynthesis of D-glucosamine-6-phosphate [69], an enzyme that has been highlighted as a potential novel target for antifungal agents [70]. The researchers synthesized eight analogs, and the compound of most interest was aminoguanidine (**48**), Figure 31. Compound (**48**) was found to have zones of inhibition of 36 mm, 25 mm, and 18 mm, respectively, for strains of *Candida albicans*, *Aspergillus oryzae*, and *Aspergillus niger*. The MIC values for (**48**) were generated for *Candida albicans*, and *Aspergillus oryzae*, and were shown to be 4 μg/mL for both. The cytotoxicity of (**48**) was evaluated in two cell lines, and the selectivity indices were found to be 12.49 and 4.42, indicative of modest selectivity. The *in vitro* IC_50_ value for the inhibition of Glc-N-6-P synthase by (**48**) was found to be 3.47 μM. This paper is valuable as it describes inhibitors of an alternate target to the currently available therapies. The activity of (**48**) is only modest, but as only a small number of analogs were described, it would make sense to synthesize additional analogs, particularly around variations on, and replacements for, the biphenyl moiety.

**Figure 32:**
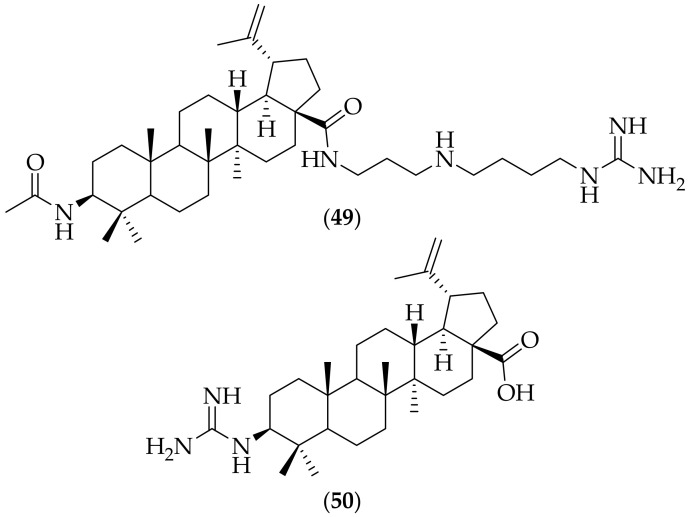
Guanidine-containing betulinic acid derived antifungal analogs (**49**) and (**50**).

Derivatives of the steroids betulinic acid, ursolic acid, and oleanolic acid were reported as having antifungal activity by Spivak et al. in 2020 [71]. Thirty four amino- and guanidine-functionalized analogs were prepared, and of these, two guanidine-containing analogs of betulinic acid were highlighted, (**49**) and (**50**), Figure 32. (**49**) was found to be inactive against *Candida auris*, but had an MIC of <0.25 μg/mL against *Cryptococcus neoformans*. (**50**) was found to have MIC’s of <0.25 μg/mL against *Candida auris*, and *Cryptococcus neoformans*. Both compounds were tested for cytotoxicity in both HEK293 cells and human red blood cells, and had CC50 values of >32 μg/mL, indicating selectivity for the fungal strains. It is curious that (**49**) was highly active against *Cryptococcus neoformans* but was inactive against *Candida auris*. Given the high potency for (**50**) it would be very interesting to have this analog tested in *in vivo* studies against *Candida auris* and *Cryptococcus neoformans* infections, and also to determine the mechanism of action for this compound.

**Figure 33:**
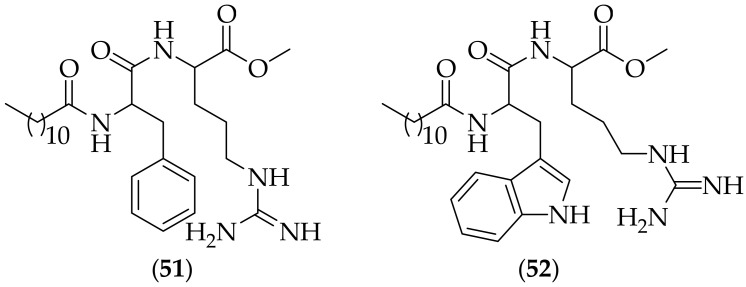
Surfactants (**51**) and (**52**) containing guanidine groups as antifungal agents.

Perez and coworkers described their synthesis and biological evaluation of two guanidine-containing surfactants in 2021, [72]. The two surfactants synthesized were (**51**), and (**52**), Figure 33, and these were evaluated using multiple methods. The two compounds were assayed for their antifungal activity against nine *Candida* strains, and both compounds displayed MIC values of 8.12 μg/mL for all nine strains, appreciably less active than the positive controls fluconazole and amphotericin B. It is very surprising that the MIC values for all nine strains were exactly the same. The compounds were next evaluated for their effect in combination with amphotericin B, and synergy was observed for both compounds against all nine *Candida* strains. An alkaline version of the comet assay was utilized to detect breaks in DNA strands in a fluconazole resistant strain of *Candida*. At MIC concentration and 2 × MIC concentration, no significant damage to the cell DNA were observed, leading the researchers to conclude that genotoxicity is not the main mechanism of action for the two compounds. Investigations in to cell viability of three *Candida* species (*C. tropicalis*, *C. albicans*, and *C. parapsilosis*) suggested that both surfactants disrupt cell membranes. Treatment of the three *Candida* species with low concentrations of either surfactant generated only low amounts of reactive oxygen species (ROS), however at concentrations 4 x MIC there was a considerable increase in ROS production. The two surfactants were tested for their ability to destroy biofilms on the same three *Candida* species, and were found to dislodge biofilms of *C. albicans* and *C. tropicalis* at 80% at a concentration of 10 x MIC, with lower activity against *C. parapsilosis*. The compounds were also shown to potentiate the antibiofilm activity of amphotericin B. The two surfactants were tested for their ability to cause hemolysis, with HC_50_ values found to be eight times higher than the MIC values. Finally, the cytotoxicity of the surfactants for L929 mammalian cells was tested, with the finding that 80–100% of cells survived at 3 x MIC concentration. Here, the two surfactants are continuing the trend of guanidine-containing agents acting as membrane disruptors. The two compounds both incorporate a methyl ester, and it is quite possible that this group will not be metabolically stable- this should be investigated prior to additonal studies being carried out with these agents.

**Figure 34:**
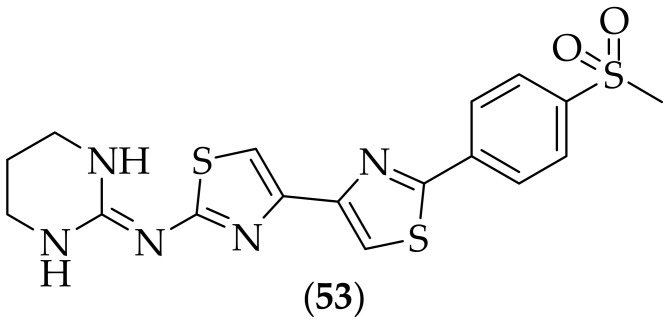
Guanidine-containing bis-thiazole analog antifungal agent (**53**).

Furthermore, in 2021, Kato et al. published their research in to thiazoyl guanidine derivatives as antifungal agents, [73]. The group initiated their investigations from the previously described thiazole abafungin (**20**), Figure 11. Detailed structure–activity relationships were reported, with discoveries including that the six-membered cyclic guanidine was essential for antifungal activity against *Aspergillus fumigatus*. From the twenty five analogs synthesized, the compound that was highlighted was (**53**), Figure 34. Compound (**53**) was not the most potent analog, but showed good potency (*A. fumigatus* MIC: 2 μg/mL), along with a lower potency shift in the presence of bovine serum (BS) (*A. fumigatus* with BS MIC: 4 μg/mL), and improved metabolic stability compared to related analogs. (**53**) was tested in an *in vivo* systemic *Aspergillus fumigatus* experiment in mice. Mice were treated iv with seven doses of the test compound over a period of 50 h, and it was shown to display higher efficacy than abafungin, at the same dose (30 mg/kg). The mode of action of (**53**) was determined to be the same as for abafungin- inhibition of ergosterol biosynthesis via inhibition of SMT (sterol 24-C-methyltransferase). Compound (**53**) was additionally shown to be active against azole-resistant strains of *Aspergillus fumigatus*, having the same MIC (2 μg/mL) for both the azole-susceptible and pan-azole-resistant strains. With the high activity previously described for abafungin, it makes good sense to work on analogs of this compound, and (**53**) looks to be a very intersting derivative, with higher *in vivo* efficacy than the parent molecule. A useful variety of analogs were prepared, but the presence of a methanesulfonyl group in (**53**) has the potential to be metabolically unstable.

**Figure 35:**
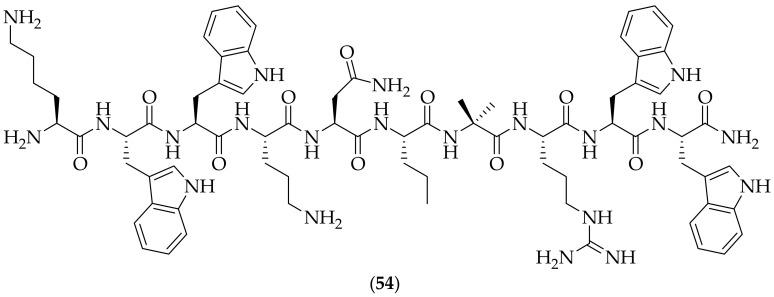
Optimized antifungal polypeptide K-oLBF127 (**54**).

Antifungal work on a one-bead, one-compound combinatorial library was published by Lam and coworkers in 2022, [74]. The group were seeking to discover membrane-active peptides, which have been described as having antifungal activities, [75,76]. Four one-bead, one-compound linear peptide libraries (8-, 10-, 12-, and 14-mer) were synthesized from 16 canonical and 8 non-canonical amino acids. Approximately 100,000 beads were immobilized, and tested for their ability to differentiate their activity between fungal and mammalian giant unilamellar vesicles, which mimic fungal and mammalian membrane compositions. From this screening a 12-mer peptide named LBF127 was discovered which displayed a six-fold selectivity between *Saccharomyces cerevisiae* and HEK293T cells. Work focused on derivatives of LBF127, and truncation of this polypeptide resulted in the discovery of polypeptide oLBF127, which has three fewer amino acids than LBF127, and is still active. A lysine group was incorporated on to the N-terminus of oLBF127, so as to improve solubility, which gave the compound focused upon in the paper, K-oLBF127 (**54**), Figure 35. (**54**) was shown to possess MIC values of as high as 5.5 μM for a variety of fungal strains, including *Cryptococcus neoformans* and *Candida tropicalis* and to have a therapeutic index of 36.4. (**54**) was shown to affect membrane integrity, through experiments utilizing trypan blue and propidium iodide. An *in vivo* efficacy study using (**54**) was carried out upon mice infected with *Cryptococcus neoformans*. The mice were dosed via intraperitoneal injection three times a day with 16 mg/kg, and the fungal burden as measured in the liver was statistically significantly reduced. However, the body weights of the treated mice were significantly lowered, suggestive of a toxic effect. This observation suggests that this polypeptide is not one that can be developed further, but given the good activity observed, additional analogs appear to be warranted to attempt to maintain the antifungal activity and remove the toxicity.

**Figure 36:**
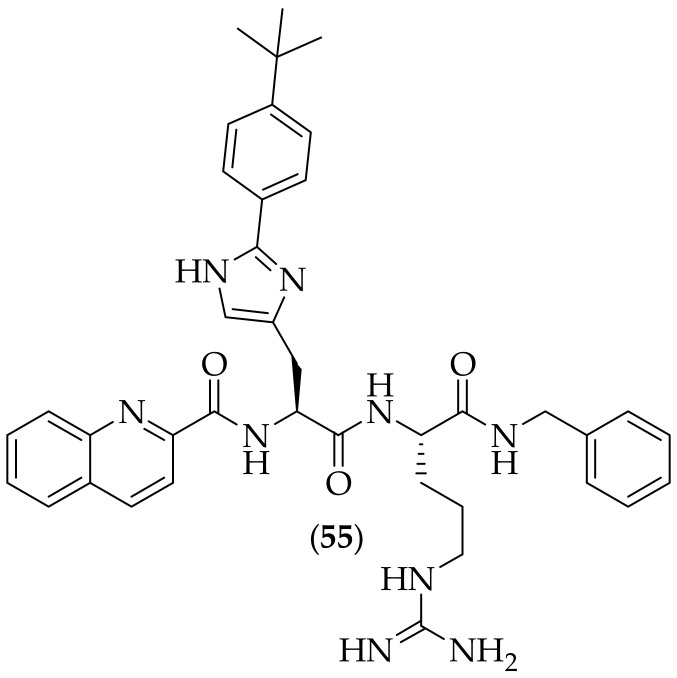
Peptide-heterocycle conjugate antifungal derivative (**55**).

Furthermore, in 2022, Jain et al. reported their work on peptide-heterocycle conjugates for the treatment of cryptococcosis, [77]. A total of twelve analogs were synthesized, with the derivatives being based upon a histidine-arginine backbone. The compound of highest interest was (**55**), Figure 36, in particular as it displayed the highest activity against *Cryptococcus neoformans* (IC_50_: 2.13 μM; MIC: 2.5 μg/mL), although this was lower than the activity measured for amphotericin B (IC_50_: 0.69 μM; MIC: 1.25 μg/mL). Compound (**55**) was additionally found to be non-cytotoxic. Analog (**55**) has only been tested against one fungal strain, and a broadening of screening to include both yeasts and molds to determine its breadth of activity would be welcome to understand how interesting this compound is.

**Figure 37:**
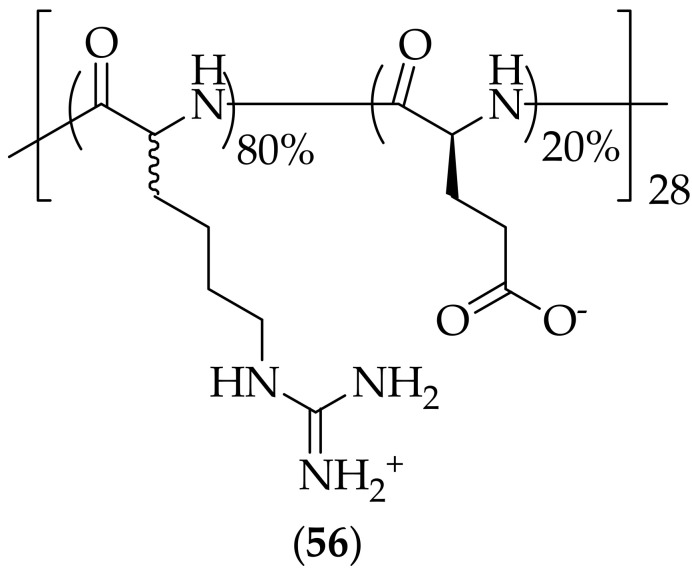
28-Mer antifungal polymer (**56**).

Improvements in cell penetrating peptides as antifungal agents were published by Liu and coworkers in 2022 [78]. Starting from a cell penetrating peptide possessing poor antifungal activity, the group first increased the chain length and homologated the amino acid, from an eight-mer (octoarginine) to a twenty eight-mer [poly(D,L-homoarginine)], which resulted in improved antifungal activity, but the new polypeptide had high cytotoxicity and precipitation issues. The scientists then incorporated an L-glutamic acid in to the polymer (shielding the positive charge density and introducing partial zwitterions), resulting in (**56**), Figure 37. This polypeptide was tested against seven strains of *Candida albicans*, and three strains of *Cryptococcus neoformans*. Both MIC and MFC values were generated for all strains, with the results showing that for *Candida albicans* the MIC and MFC values ranged from 1.56–6.25 μg/mL, and for *Cryptococcus neoformans* the MIC values were 0.78 μg/mL, and the MFC values were 1.56 μg/mL. Polymer (**56**) was found to show negligible hemolysis of human red blood cells (RBCs), with an HC_10_ of >2000 μg/mL. Several types of mammalian cells were also subjected to (**56**), with the results showing that the polypeptide had low cytotoxicity (IC_50_ = 400 μg/mL). The ability of *Candida albicans* to develop resistance to (**56**) was evaluated, and it was discovered that no resistance was generated after 24 passages of treatment. Polypeptide (**56**) was tested for its aqueous solubility, and was found to have high solubility of >500 mg/mL. The mechanism of action for (**56**) was probed using a green-fluorescent dye-conjugated derivative of (**56**), and the results when *Candida albicans* cells were incubated with this dye-conjugted derivative and propidium iodide suggested that the polymer entered cells. Additional investigation showed that there was a mild interaction between (**56**) and the fungal membrane. Scanning electron microscopy was utilized to evaluate the effect of (**56**) on *Candida albicans*, and it was shown that “small individual pits or pores on cell membranes” developed upon exposure to (**56**), which was consistent with the mild membrane interaction previously described. Furthermore, found was damage to the organelle structures of mitochondria. The studies overall suggested that the antifungal mechanism of the polypeptide was that the agent penetrates the fungal cell membrane, and induces organelle destruction within the cells. An *in vivo* systemic *Candida albicans* murine experiment was carried out with (**56**), dosing at 15 mg/kg once a day over a period of 6 days. The results showed that there was an 85% survival rate in the treated mice. The fungal burdens in the major organs of the mice showed that treatment with (**56**) resulted in 1–2 log reduction in fungal cell densities compared to saline-treated mice. Polymer (**56**) is highly interesting, with excellent MIC and MFC values, along with information on its mechanism of action, and *in vivo* efficacy.

**Figure 38:**
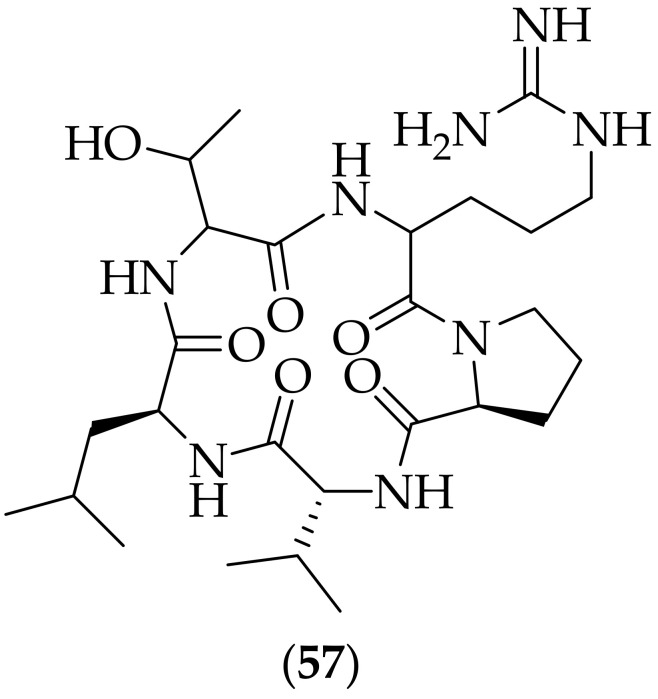
Antifungal cyclic pentapeptide (**57**).

Four cyclic polypeptides were described as antifungal agents by Jadeja et al. in 2022 [79]. The analogs were synthesized using a head-to-tail cyclization process, which was predicted to decrease the polarity of the analogs, relative to the linear polypeptide, and thus increase the cell membrane permeability. Of the four cyclic polypeptides synthesized, the one highlighted was (**57**) [cyclo(threonine-arginine-proline-D-valine-leucine)], Figure 38. Cyclic pentapeptide (**57**) was tested against strains of *Aspergillus flavus*, *Candida albicans*, and *Candida glabrata*. (**57**) was found to be fungicidal for all three strains, with MIC values of >80–150 mg/mL, comparable to the reference standard antifungal agents employed, amphotericin B and miconazole. The scientists carried out molecular docking studies with the four polypeptides for their interactions with lanosterol 14-α demethylase (the target of the azole class of antifungal drugs such as voriconazole [80]), and identified a correlation between biological activity and docking scores for the four peptides. Given the relatively low MIC values found for analog (**57**), for such cyclic polypeptides to be able to progress further it would seem that additonal analogs need to be synthesized and tested to discover more potent derivatives.

**Figure 39:**
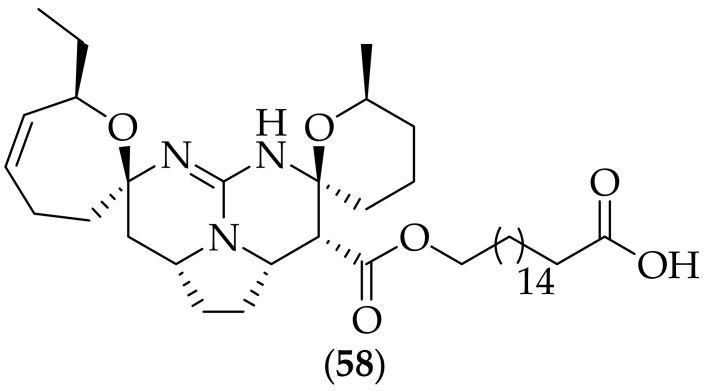
Structure of crambescidic acid-671 (**58**).

Furthermore, in 2022, Wanas et al. published their research in to alkaloids isolated from the dichloromethane/methanol extract of the marine sponges *Monanchora clathrata* and *Monanchora unguiculata*, [81]. The four alkaloids isolated from *Monanchora clathrata* were found to have potent antifungal activity, whilst three of the four alkaloids isolated from *Monanchora unguiculata* were found to have potent antifungal activity (the three active compounds from *Monanchora unguiculata* were three of the four alkaloids isolated from *Monanchora clathrata*). Of the four active compounds, the most potent was the novel guanidine alkaloid crambescidic acid-671 (**58**), Figure 39 (a derivative of crambescidic acid, which was originally isolated from the marine sponge *Monanchora unguifera* [82])- the antifungal activities of (**58**) were, *Candida albicans* MIC: 5.0 μg/mL, *Aspergillus fumigatus* MIC: 5.0 μg/mL, *Crpytococcus neoformans* MIC: 0.16 μg/mL; MFC 0.16 μg/mL, and *Cryptococcus gattii* MIC: 0.16 μg/mL; MFC 0.16 μg/mL. These antifungal potencies were comparable to the positive control amphotericin B against *Aspergillus fumigatus*, *Cryptococcus neoformans*, and *Cryptococcus gattii*. With the high activity of natural product (**58**) against the two *Cryptococcus* species, it would have been instructive for the authors to have tested the compound in an *in vivo* cryptococcosis experiment to discover if the agent could be beneficial for this indication.

**Figure 40:**
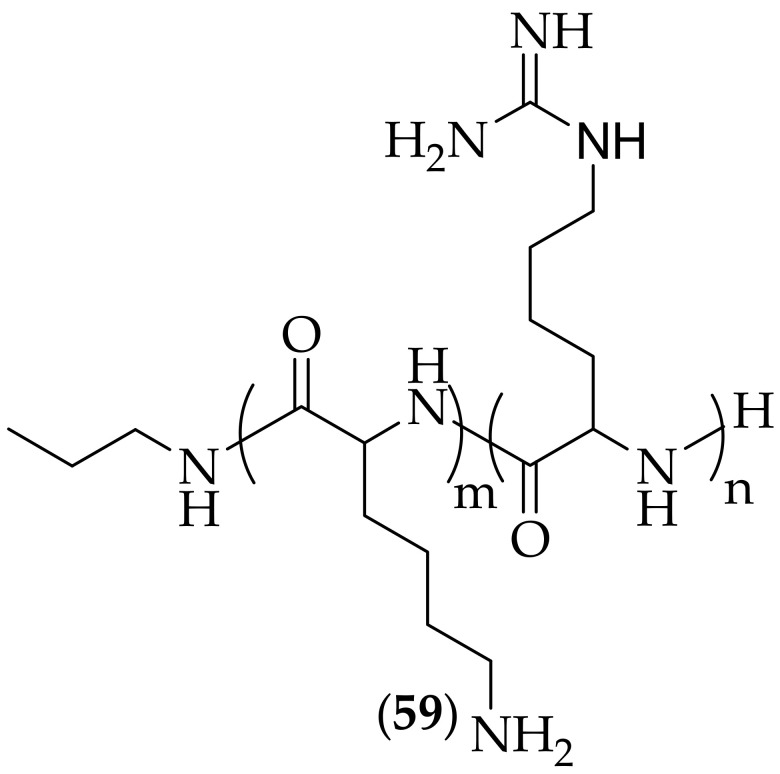
General structure of guanylated lysine-based polymer (**59**).

Ji and co-workers published their discovery of the benefical effect on antifungal potency of amino guanylation on a lysine-based polymer in 2022, [83]. The scientists initially synthesized a set of four antimicrobial peptide-mimicking polymers based on a poly-lysine template (with number average molecular weights of 1700, 2500, 2900, and 3300 g/mol), and found that the polymers were not active as antifungal agents against *Candida albicans*, *Candida krusei*, *Candida glabrata*, and *Candida parapsilosis*, but had an MIC of <3 μg/mL against *Candida tropicalis*. The polymers were then derivatized with varying percentages of guanylaion (20–100%), resulting in eight novel analogs, with the reasoning for this change being that it is known in the literature that guanylation can have a beneficial effect on the antifungal activity of antimicrobial-mimicking polymers [84,85,86,87]. The most interesting of the analogs was termed α-PL3-G100, where the G100 value indicates 100% guanylation, general structure (**59**), Figure 40. This analog displayed antifungal activity against all five fungal species tested against; *Candida albicans* MIC: 48 μg/mL, *Candida krusei* MIC: 24 μg/mL, *Candida glabrata* MIC: 1500 μg/mL, *Candida parapsilosis* MIC: 96 μg/mL, and *Candida tropicalis* MIC: <3 μg/mL. The HC_10_ hemolytic activity of α-PL3-G100 was calculated to be >3000 μg/mL, resulting in a selectivity index of >62 for *Candida albicans*. The cytotoxicity of the polymers was assessed using an MTT assay against NIH 3T3 mouse embryonic fibroblast cells over 24 h. The results showed that the polymers became more toxic to cells as the molcular weight increased, with IC_50_ values for the guanylated polymers of ~50 μg/mL (in the same range as the antifungal potencies observed). Scanning electron microscopy was utilized to investigate morphological changes of *Candida albicans* treated with α-PL3-G100. At a concentation of 4 X MIC for 4 h, a large fraction of the *Candida albicans* treated with α-PL3-G100 “wrinkled, and some of them largely deformed or even had deep hollows”, whereas “untreated *Candida albicans* had a clear, smooth surface”. These effects were interpreted to indicate that the polymer caused damage to *Candida albicans* through membrane disruption. The damage caused to *Candida albicans* cell membranes was further investigated using propidium iodide, and it was found that upon treatment with α-PL3-G100, red fluorescence was observed, indicative of membrane disruption. This polymer is another example of guanidine-containing antifungal agents acting as membrane disruptors. The compound only displays low antifungal activity versus the strains tested, with the exception of *Candida tropicalis*. It is not clear for a path forward for this compound.

## 2. Conclusions

As can readily be discerned, there have been a plethora of guanidine-containing compounds disclosed between 2004 and 2022 as having antifungal activity against human related fungal pathogens, both *in vitro* and *in vivo*. Given the strong basicity of many of the guanidines in the molecules, it can be postulated that this physical property is beneficial for the production of antifungal activity, possibly in certain cases by means of interaction with the negatively charged fungal cell wall (corroborated by the fact that multiple guanidine-containing agents described herein act through cell wall interaction). It is noted that for many of the antifungal agents there is no cytotoxicity data associated with the compounds, and it would be instructive to have this data for more derivatives, so as to be able to discount toxicity as a means of effecting antifungal potency. The majority of the publications also do not include details on the mechanism of action of the compounds, and this would be highly benefical information. It would also be useful for there to be greater publication of *in vivo* efficacy experiments with guanidine-containing derivatives so that a stronger body of work can be shown. The *in vivo* experiments that have been described on guanidine-containing antifungal derivatives between 2004 and 2022 are summarized in Table 1. Thus, it has been shown that macrocycles (**11**), semi-synthetic analogs (**39**), small molecules [(**45**) and (**53**)], polypeptides (**34**), and (**54**), and polymers (**56**) all display *in vivo* efficacy. Among these, perhaps the most interesting agents are macrocycle BM1 (**11**), abafungin-related analog (**53**), and polymer (**56**), due to the large amount of positive data collected for each compound, coupled with *in vivo* antifungal efficacy. Another agent of high interest is (**48**), a GlcN-6-P synthase inhibitor, as this agent is known to have a novel mechanism of action. It is hoped that in time there will be a guanidine-containing analog that progresses in to clinical evaluation for antifungal treatment, and that one day there will be an approved antifungal agent that contains a guanidine functionality which can benefit patients with fungal diseases, perhaps (**11**), (**34**), (**53**), or (**56**).

## Figures and Tables

**Table 1:** Summary of the *in vivo* efficacy of guanidine-containing derivatives.

Compound	Reference	Fungal Species	Dose
Cyclised guazatine-related analog (**11**)	[23]	*Candida albicans*	20, 40 mg/kg
Short cationic antimicrobial peptide (**34**)	[45]	*Candida albicans*	2% hydrogel
α-Mangostin derived analog (**39**)	[52]	*Fungal keratitis*	0.2% solution
Thiazole-aminoguanidine analog (**45**)	[67]	*Candida albicans*	5 μg/mL
Thiazole-aminoguanidine analog (**45**)	[67]	*Candida auris*	10 μg/mL
Abafungin-derived (**53**)	[73]	*Aspergillus fumigatus*	30 mg/kg
Polypeptide K-oLBF127 (**54**)	[74]	*Cryptococcus neoformans*	16 mg/kg
Antifungal polymer (**56)**	[78]	*Candida albicans*	15 mg/kg

## Data Availability

Not applicable.
